# A Similar Nonclinical Safety Evaluation of Prev(e)nar 13 in a Multi-Dose Formulation Containing the Preservative 2-Phenoxyethanol

**DOI:** 10.3390/vaccines13050486

**Published:** 2025-04-30

**Authors:** Yana Chervona, Wen Shen, Shambhunath Choudhary, Victoria Markiewicz, Peter C. Giardina, Cynthia M. Rohde

**Affiliations:** 1 Drug Safety Research and Development, Pfizer Worldwide Research, Development & Medical, Pfizer, Inc., Pearl River, NY 10965, USA; shambhunath.choudhary@pfizer.com (S.C.); peter.giardina@pfizer.com (P.C.G.); cynthia.rohde@pfizer.com (C.M.R.); 2 ADARx Pharmaceuticals, Inc., San Diego, CA 92121, USA; wshen@adarx.com; 3 Drug Safety Research and Development, Pfizer Worldwide Research, Development & Medical, Pfizer, Inc., Groton, CT 06340, USA; vmeasternct@gmail.com

**Keywords:** 13vPnC, multi-dose vial (MDV), phenoxyethanol (2-PE), rabbit, toxicity, nonclinical safety

## Abstract

Background: 2-Phenoxyethanol (2-PE) has been safely included as a preservative and/or stabilizer in more than thirty vaccine formulations at amounts ranging from 0.5 to 5 mg per dose; however, the nonclinical safety data publicly available for intramuscular (IM) or subcutaneous (SC) administration are relatively limited. Here, in addition to the available clinical and nonclinical data for 2-PE, we summarize the nonclinical safety data of experimental 13vPnC (Prev(e)nar 13) formulations with or without 2-PE. Methods: Two repeat-dose toxicity studies in rabbits, one for a 2-PE-free formulation of 13vPnC and the other for an MDV formulation of 13vPnC with 5 mg/dose 2-PE, were conducted as part of an overall nonclinical safety package for vaccine development. The studies were designed and conducted in compliance with the relevant guidelines and regulations. Results: In repeat-dose toxicity studies in rabbits, five IM administrations of a preservative-free 13vPnC single-dose syringe formulation or a 13vPnC multi-dose vial (MDV) formulation containing 5 mg 2-PE/0.5 mL dose were well tolerated with no systemic toxicity. Robust serotype-specific IgG antibody responses to each of the 13 pneumococcal serotypes were also confirmed for both formulations. The observations for the 13vPnC MDV including local inflammatory reaction, increases in fibrinogen, and increased splenic germinal centers were nonadverse, reversible, and consistent with findings previously observed for the IM administration of vaccines, including the 2-PE-free 13vPnC single-dose syringe formulation. Conclusions: Together with the other available nonclinical and clinical data of 2-PE and vaccine formulations containing 2-PE and following the 3Rs principle, our risk-assessment-based recommendation is that no additional nonclinical safety studies are needed when evaluating a 2-PE-containing presentation of a previously well-characterized vaccine product if the amount of 2-PE is ≤10 mg/dose.

## 1. Introduction

*Streptococcus pneumoniae* (pneumococcus) is a Gram-positive bacterium that is responsible for a wide range of upper and lower respiratory tract diseases, including pneumonia, otitis media, septicemia, meningitis, and sinusitis [1,2]. This opportunistic pathogen is thought to initially colonize the mucosal surfaces of the nasopharynx and then progress to the lower parts of the respiratory tract, resulting in systemic disease [1]. As recently as 2021, pneumococcus was the leading cause of both morbidity and mortality among lower respiratory tract infections globally, resulting in approximately 97.9 million (95% UI 92.1–104.0) episodes of lower respiratory infections (LRIs) and causing an estimated 505,000 deaths (95% UI 454,000–555,000) [3].

Prev(e)nar 13 is a 13-valent pneumococcal conjugate vaccine composed of saccharides of the capsular antigens of *Streptococcus pneumoniae* serotypes 1, 3, 4, 5, 6A, 6B, 7F, 9V, 14, 18C, 19A, 19F, and 23F, individually linked to non-toxic diphtheria CRM197 protein isolated from *Corynebacterium diphtheria* [4]. Prev(e)nar 13, recommended for the active immunization of infants, toddlers, and adults to provide broad serotype coverage against *S. pneumoniae*, was approved in the EU and the U.S. in 2009 and 2010, respectively. In 2016, the World Health Organization granted prequalification for multi-dose vial (MDV) presentation of Prev(e)nar 13, aiding in increasing the availability of the vaccine for children in resource-limiting countries. The Prev(e)nar 13 MDV presentation includes the preservative 2-PE at 4 mg/dose, which enables the use of the vaccine over a 28-day period following its first use when stored at 2–8 °C. 2-PE was reported to provide a superior antimicrobial effectiveness over thimerosal, another vaccine preservative, for Prev(e)nar 13 in both single- and multi-challenge preservative effectiveness studies [5]. Prev(e)nar 13 formulations containing 2-PE between 3.5 and 5.5 mg/dose were stable and met European Pharmacopoeia (EP)-recommended criteria B for antimicrobial preservative effectiveness [5]. The Prev(e)nar 13 MDV was found to be safe and non-inferior to the preservative-free single-dose syringe formulation of Prev(e)nar 13 in clinical trials in infants [6,7].

2-PE (CAS number: 122-99-6) is a broad-spectrum preservative used in pharmaceutical products against a wide range of Gram-negative and Gram-positive bacteria and yeasts [8]. In vaccine formulations, for example, 2-PE has been used as a preservative or stabilizer at levels ranging from 2.5 to 5 mg/dose (Table 1, Table 2 and Table 3), while in topical drug products it as has been used at concentration of up to 2.2% [9].

2-PE is rapidly absorbed, distributed, metabolized, and excreted in urine mainly as phenoxyacetic acid (2-PAA) in nonclinical species and humans, independent of the administration route [9]. 2-PE is primarily metabolized in the liver, and the rate of metabolism of 2-PE to 2-PAA in vitro is the highest in humans, followed by that in rats, mice, and rabbits. These data suggest that the metabolizing enzymes may be saturated at a lower concentration of 2-PE in livers of rabbits than that in other species, indicating that the rabbit is a more sensitive as a species to 2-PE than humans, rats, and mice [9].

Most nonclinical toxicology studies evaluated the effects of 2-PE following oral or dermal administration instead of routes that are typical for vaccine administration (IM or SC) [9]. These studies were thoroughly reviewed by the Scientific Committee on Consumer Safety (SCCS) in 2016 [9]. The nonclinical data of 2-PE following oral administration are of limited relevance to understand 2-PE safety via IM or SC vaccine administration due to extensive first-pass metabolism leading to a very low systemic availability of 2-PE (0.07% and 1.6% of the bioavailable dose in rats and rabbits, respectively) [9]. However, following dermal administration, the bioavailability of 2-PE was ~78–81% [9,10], suggesting that the dermal-route nonclinical and/or clinical data may be more relevant to understand the potential toxicity associated with the parenteral administration of 2-PE.

Although 2-PE has been safely used in more than 30 single- or multi-dose vaccine formulations as a preservative or stabilizer (Table 1, Table 2 and Table 3), the nonclinical safety data for vaccines containing 2-PE are relatively limited. One notable exception is the publication of the nonclinical developmental toxicity data for Adacel, a tetanus, diphtheria, and pertussis (Tdap) vaccine [11]. Adacel contains 2-PE at 3.3 mg/0.5 mL dose (as a stabilizer). There were no vaccine-related effects on pregnancy, parturition, lactation, pre- or post-natal development, or fetal malformations in rabbits administered three full single human doses of Adacel.

To further enrich the nonclinical safety profile of 2-PE, in addition to the clinical safety data associated with 2-PE administration alone or as part of vaccine formulations, we summarize the results from two repeat-dose toxicity studies in rabbits: one for a 2-PE-free formulation of 13vPnC (Prev(e)nar 13) and the other for an MDV formulation of 13vPnC with 5 mg/dose 2-PE and provide a risk-assessment based recommendation for not conducting additional nonclinical safety studies when evaluating a 2-PE-containing presentation of a previously well-characterized vaccine product if the amount of 2-PE is ≤20 mg/mL or ≤10 mg/dose.

**Table 1:** U.S.-approved vaccines containing 2-PE.

Vaccine	Age Group	Vaccine Type	2-PE Dose	Route/ Dose (mL)	Company
IPOL [12]	6 weeks and older	IPV	0.50% ^a^	IM, SC/0.5	Sanofi
Adacel ^b^ [11]	10 to 64 years	Tdap	3.3 mg/dose	IM/0.5	Sanofi
Daptacel ^b^ [13]	6 weeks through 6 years	DTaP	3.3 mg/dose	IM/0.5	Sanofi
Pentacel ^b^ [14]	6 weeks through 4 years	DTaP-IPV-Hib	3.3 mg/dose	IM/0.5	Sanofi
Quadracel ^b^ [15]	4 through 6 years	DTap-IPV	3.3 mg/dose	IM/0.5	Sanofi

a. Unit of concentration (*v*/*v* or *w*/*v*) not specified. b. 2-PE is included in the listed vaccines as a stabilizer (not a preservative).

**Table 2:** Vaccines containing 2-PE that are approved outside of the U.S.

Vaccine	Age Group	Vaccine Type	2-PE Dose	Route/ Dose (mL)	Company
Prev(e)nar 13 MDV [4,16]	6 weeks and older	Pneumococcal	4 mg/dose	IM/0.5	Pfizer
Tetravac [17]	2 months to 12 years, with additional booster recommended between the ages of 4 and 13	DTaP-IPV	2.5 µL 0.5% *v*/*v* ^a^ 2.75 mg/dose	IM/0.5	Sanofi
Revaxis [18]	6 years or older	Td-IPV	NA ^b^	IM/0.5	Sanofi
Repevax [19]	3 years or older	DTaP-IPV	NA ^b^	IM/0.5	Sanofi
Avaxim (pediatric) [20]	1 to 15 years ^c^	HepA	2.5 µL 0.5% *v*/*v* ^a^ 2.75 mg/dose	IM/0.5	Sanofi
Avaxim [21,22]	12 years or older ^d^	HepA	2.5 µL 0.5% *v*/*v* ^a^ 2.75 mg/dose	IM/0.5	Sanofi
IMOVAX Polio [23]	2 months or older	IPV	≤1.0% ^e^	SC/0.5	Sanofi
ViVAXIM [24]	16 years or older	HepA-Typhoid	2.5 µL 0.25% *v*/*v* ^a^ 2.75 mg/dose	IM/1	Sanofi
Td Adsorbed [25]	7 years and older	Td	0.6% *v*/*v* ^a^ 3.3 mg/dose	IM/0.5	Sanofi
Kinrix [26]	4 through 6 years	DTaP-IPV	≤2.5 mg/dose	IM/0.5	GSK
HEXASIL [27]	6 weeks and older	DTwP-HepB-IPV-Hib	0.5% ^e^	IM/0.5	Serum Institute of India Pvt. Ltd.
Eupolio Inj. [28]	6 weeks and older	Sabin IPV	2.5 mg/dose	IM/0.5	LG Chem Ltd.
Picovax [29]	6 weeks and older	IPV	0.5% *w*/*v* 2.5 mg/dose	IM/0.5	AJ Vaccines A/S
Poliomyelitis Vaccine (Inactivated) [30]	6 weeks and older	IPV	2.5 mg/dose	IM, SC/0.5	Serum Institute of India Pvt. Ltd.
Poliomyelitis Vaccine [31]	Children and adults	IPV	5 mg/mL 2.5 mg/dose	IM, SC/0.5	Bilthoven Biologicals B.V.
ShanIPV [32]	6 weeks and older	IPV	2.5 µL/dose 2.75 mg/dose	IM, SC/0.5	Sanofi
Poliomyelitis Vaccine (Vero Cell), Inactivated, Sabin Strains [33]	2 months and older	SIPV	NA ^b^	IM/0.5	Sinovac Biotech Co. Ltd.
Synflorix [34]	6 weeks to 5 years	Pneumococcal	10 mg/mL 5 mg/dose	IM/0.5	GSK
SKYTyphoid Multi Inj. [35]	6 months to 45 years	Typhoid	5 mg/dose	IM/0.5	SK Bioscience Co., Ltd.
TYPHIBEV [36]	6 months to 45 years	Typhoid	5 mg/dose	IM/0.5	Biological E. Limited
Typbar-TCV [37]	6 months to 45 years	Typhoid	5 mg/dose	IM/0.5	Bharat Biotech International Limited
ZyVac [38]	6 months to 45 years	Typhoid	0.5 mg/dose	IM/0.5	Zydus Lifesciences Limited

a. 2-PE dose (mg/dose) = *v*/*v* concentration × 2-PE density (1.1 g/mL) × dose volume (mL) × 1000 mg/1 g. b. Information of 2-PE amount in the listed vaccine is not available. c. Avaxim pediatric is approved for use in children 1–15 years of age (inclusive) in Canada. NACI also indicates that hepatitis A vaccine may be provided, beginning at 6 months of age, to infants who are at increased risk of infection or have severe hepatitis A. d. Avaxim is indicated for the immunization of persons 12 or 16 years of age or older depending on each country. e. Unit of concentration (*v*/*v* or *w*/*v*) not specified.

**Table 3:** Marketed vaccines initially approved with a 2-PE-containing formulation (the current U.S./EU formulation no longer contains 2-PE) and discontinued 2-PE containing vaccine(s).

Vaccine	Age Group	Vaccine Type	2-PE Dose	Route/ Dose (mL)	Company
Havrix (pediatric) [39,40]	1 through 18 years	HepA	0.5% *w*/*v* ^a^ 2.5 mg/dose	IM/0.5	GSK
Havrix (adult) [39,40]	19 years or older	HepA	0.5% *w*/*v* ^a^ 5 mg/dose	IM/1	GSK
Twinrix (adult) [41,42]	16 years or older	HepA-HepB	5 mg/dose	IM/1	GSK
Pediarix [43,44]	6 weeks through 6 years	DTaP-HepB-IPV	2.5 mg/dose	IM/0.5	GSK
Infanrix Hexa [45,46]	6 weeks to 2 years	DTaP-IPV-Hib-HepB	2.5 mg/dose	IM/0.5	GSK
Discontinued 2-PE-containing vaccines					
LYMErix [47] ^b^	15 to 70 years	Lyme Disease	2.5 mg/dose	IM/0.5	GSK
Infanrix [48,49] ^b^	6 weeks through 6 years	DTaP	2.5 mg/dose	IM/0.5	GSK
Poliorix [50]	6 weeks or older	IPV	NA ^d^	IM/0.5	GSK
ViATIM [51] ^c^	16 years or older	HepA-Typhoid	NA	Slow IM, SC/1	Sanofi

a. 2-PE dose (mg/dose) = *w*/*v* concentration (g/mL) × dose volume (mL) × 1000 mg/1 g. b. Years on market: 1998–2002. c. Product no longer authorized in Europe. d. Information of 2-PE amount in the listed vaccine is not available.

## 2. Materials and Methods

### 2.1. Animals and Husbandry

All the animal care and experimental procedures were conducted in compliance with the guidelines for the care and use of laboratory animals [52] as well as local regulations. Each study was conducted according to the GLP and OECD guidelines.

Male and female New Zealand White (NZW) rabbits were supplied by Covance Research Products Inc., Denver, PA, U.S. (approximately 5 to 6 months at dosing start). Rabbits were selected as the test species because they are commonly used in vaccine toxicity studies, have a large historical database, and produce an antigen-specific immune response to pneumococcal polysaccharide conjugates.

The animals were offered food (Certified Teklad Rabbit Diet #2030C (Harlan Teklad now Envigo which is an Inotiv, Inc. company [Study 1]) or Certified Rabbit High-Fiber #5325 (PMI Nutrition International LLC [Study 2])), except when fasted prior to euthanasia, and locally sourced water ad libitum. The animals were housed individually throughout the study period in plastic (Study 1) or stainless-steel (Study 2) cages. The environmental conditions across the studies were set to maintain relative humidity ranging from 30% to 70% and temperature ranging from 60 °F to 72 °F, with the room lighting set to provide a 12 h light/dark cycle.

### 2.2. Test and Control Articles

13vPnC (Study 1) is a sterile liquid containing 4.4 μg/mL of each of the serotypes, except for serotype 6B at 8.8 μg/mL, and is formulated with a succinate buffer containing polysorbate 80 and NaCl as excipients at pH 5.8 with aluminum phosphate (AlPO_4_) at 0.25 mg aluminum/mL as an adjuvant, packaged as a single-dose syringe. The composition of the 13vPnC MDV (Study 2) is the same as that of 13vPnC with an additional 5 mg 2-PE per 0.5 mL dose. The vehicle control groups for 13vPnC and 13vPnC MDV (both studies) contained aluminum phosphate (AlPO_4_) (at 0.25 mg aluminum/mL) in succinate buffer at pH 5.8, polysorbate 80, and sodium chloride. The 13vPnC MDV vehicle control (Study 2) also included 10 mg/mL 2-PE. Sterile saline solution (0.9%) at pH 7.0 was also used as a control in Study 1.

### 2.3. Study Design

**Figure 1:**
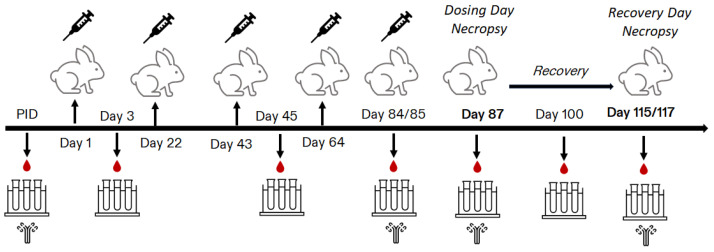
Overview of the experimental design. Male and female New Zealand White rabbits (10/sex/group) were administered 5 IM injections once every three weeks of control (saline), vehicle control, 13vPnC MDV vehicle control, 13vPnC, or 13vPnC MDV. The animals were euthanized two days after the last dose, Day 87 (5 animals/sex/group) and approximately 4 weeks later (Days 115 or 117). Blood was collected for serology analysis prior to dose initiation, on Day 84 and at each necropsy. Blood was collected for clinical pathology assessments on Days 3 and 45 and at each necropsy.

Repeat-dose toxicity studies were conducted to support the development of 13vPnC and 13vPnC MDV. The first study was conducted at Wyeth Research (now Pfizer, Inc.), Drug Safety, Chazy, NY, USA (Study 1). The second study was conducted at Covance Laboratories Inc. (now Labcorp), Madison, WI, USA (Study 2). Both the studies had a similar design (Figure 1).

Briefly, male and female NZW rabbits (10/sex/group) were acclimated, randomly assigned to groups, and then administered control or test article material (1 dose every 3 weeks, on dosing-phase Days 1, 22, 43, 64, and 85) via IM administration, which was also the route of administration at the clinic. A subset of animals (5/sex/group) was euthanized (anesthetized with sodium pentobarbital and then exsanguinated) 2 days after the final dose (Day 87), while the remaining animals (5/sex/group) underwent an approximate 4-week recovery and then were euthanized (Study 1: Day 115; Study 2: Day 117). In Study 1, the rabbits were administered saline control, vehicle control, or 13vPnC. In Study 2, the rabbits were administered vehicle control, 13vPnC MDV vehicle control, or 13vPnC MDV. Saline was not used as a control for Study 2. The doses and dose volumes in both the studies were the same as the clinical dose and dose volume (0.5 mL per injection). The IM injections in both the studies were administered into the quadriceps femoris muscle of the right hind leg.

### 2.4. In-Life Assessments

Clinical signs, body weight, and food consumption were monitored throughout this study. Ophthalmologic examinations were performed once during the pre-dose phase, at the end of the dosing phase, and at the end of the recovery phase. On the days of dosing, the injection sites were evaluated for irritation using the Modified Draize Method [53] prior to dosing and approximately 4, 24, 48, and/or 72 h post-dose. The injection sites were also evaluated on the day of recovery necropsy (Day 115), prior to euthanasia (Study 1) and 24 HPD the last dose (Day 86) (Study 2). The body temperature of each animal was recorded prior to dosing and approximately 4 and 24 h post-dosing on the dosing day (Study 1 and 2) and on the day of recovery necropsy (Day 115; Study 1 only).

### 2.5. Blood Sample Collections for Clinical Pathology

The hematology parameters (including hematology parameters and coagulation parameters, Supplemental Table S1) and clinical chemistry parameters (Supplemental Table S2) were examined once pretest, during the dosing phase (on Days 3, 45, and 87), and during the recovery phase (Study 1: on Days 100 and 115, Study 2: Day 117).

### 2.6. Post-Mortem Assessments

At the dosing- and recovery-phase necropsies, the terminal body weights, organ weights, and macroscopic and microscopic observations were recorded. Representative samples of the organs and tissues listed in Supplemental Table S1 were fixed and examined microscopically.

### 2.7. Serology Analysis

For Study 1, rabbit serum samples were obtained at pre-dose, at dosing-phase Days 42 and 84 for serology analysis, and on the days of scheduled necropsy for serology analysis (from animals designated for necropsy). For Study 2, the rabbit serum samples were obtained at pre-dose, at dosing Days 84 and 87 (dosing-phase necropsy day), and on the day of scheduled recovery necropsy (Day 117). The serum samples were analyzed for IgG antibody concentrations specific to each of the 13 pneumococcal serotypes included in the 13vPnC test article, using a Luminex-based 13-valent fluorescent microsphere-based immunoassay assay method (13-plex IgG dLIA). For Study 1, only the pre-dose and dosing Day 84 samples were analyzed, and, for Study 2, all the serology timepoints were analyzed.

### 2.8. Statistical Analysis

Statistical analyses of the body weight, body temperature, hematology, clinical chemistry, urinalysis, relative and absolute organ weights, and injection-site scores were conducted. An analysis of body weight was performed at the last pretest and at all the following sampling times. As previously described elsewhere [54], the analysis of body temperature was based on the maximum body temperature post-injection for each animal. The analysis of the injection-site score was based on the average irritation score post-injection for each animal. A nonparametric (rank-transform) one-way analysis of variance (ANOVA) was conducted, with a two-sided pairwise comparison of each dose group to the reference group (vehicle control) using Dunnett’s test. Average ranks were assigned to ties. The analyses were carried out separately for each sex.

## 3. Results

### 3.1. In-Life Findings

In both the repeat-dose toxicity studies, the IM administration of vaccine candidates (13vPnC or 13vPnC MDV) was tolerated without evidence of systemic toxicity. There were no vaccine-related mortalities or effects on clinical signs, food consumption, body weights, body temperature, ophthalmology parameters, or injection-site scores.

### 3.2. Clinical Pathology

**Figure 2:**
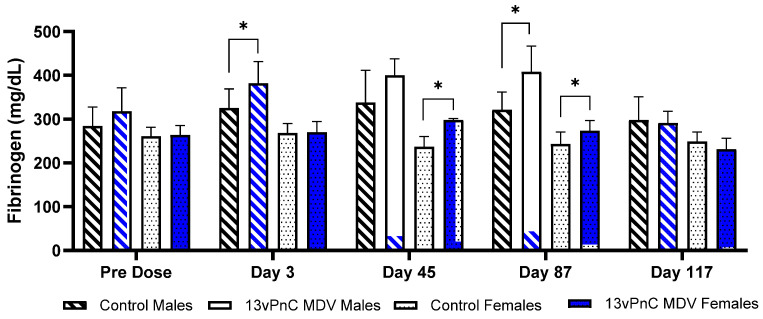
Fibrinogen concentrations in male and female New Zealand White rabbits immunized IM with 13vPnC MDV or vehicle control. Blood samples were collected on Days 3, 45, 87, and 117. Statistical comparisons between control and immunized animals were performed using a nonparametric (rank-transform) one-way ANOVA with a 2-sided pairwise comparison of each dose group to the reference group (vehicle control) using Dunnett’s test (* *p* < 0.05).

There were no clinical pathology changes in Study 1 (13vPnC). In Study 2, minimal increases in the fibrinogen concentration were present in animals administered 13vPnC MDV during the dosing phase on Day 3 in males, on Day 45 in females with a similar trend in males, and on Day 87 in both sexes (Figure 2). These minor, nonadverse changes in fibrinogen concentration were reflective of the acute-phase response to the vaccine and completely reversed by Day 29 of the recovery phase. Administration of the 13vPnC MDV vehicle control had no effect on the fibrinogen concentrations.

### 3.3. Organ Weight/Macroscopic and Microscopic Observatoins

The microscopic findings at the injection site and in the spleen of the animals administered vehicle control, 13vPnC MDV vehicle control, 13vPnC, and/or 13vPnC MDV were attributed to AlPO_4_ (Table 4 and Table 5).

Chronic inflammation and degeneration/necrosis of skeletal muscle myofibers at the injection site were noted in both the studies. In Study 1, minimal-to-moderate chronic inflammation and degeneration/necrosis of myofibers was noted at the injection sites in test-article-treated animals compared with minimal-to-mild chronic inflammation and degeneration/necrosis of myofibers in vehicle-control animals; the increase in severity and/or incidence among the vaccinated groups was consistent with an expectant inflammatory response to the antigens in the vaccine. In Study 2, the incidence and severity of chronic inflammation and degeneration/necrosis of myofibers was comparable across the study groups. The degeneration/necrosis of myofibers was characterized by vacuolation and/or lysis. Regenerative fibers were occasionally present. Chronic inflammation was characterized by infiltrates of macrophages, heterophils, and lymphocytes in variable numbers and combinations. The macrophages often contained prominent basophilic cytoplasmic material, which is characteristic of AlPO_4_. Inflammation occurred primarily in the interstitium between the myofibers of the skeletal muscle but was also occasionally localized in the overlying skin. In Study 2, an additional microscopic change included minimal increased germinal centers in the spleen of animals given the 13vPnC MDV. The microscopic changes noted in both the studies were not adverse as there was no effect on the general health of the animals or evidence of systemic toxicity and full-to-partial recovery was observed based on a decrease in incidence and/or severity.

### 3.4. Serology

**Figure 3:**
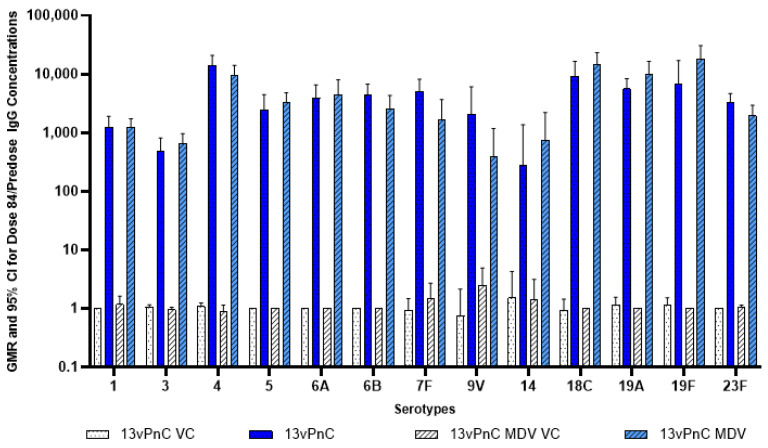
New Zealand White rabbits were immunized IM with 13vPnC, 13vPnC MDV, vehicle control (13vPnC VC), or 13vPnC MDV vehicle control (13cPnC MDV VC). Analysis of serum samples was performed pre-dose (Dosing Day 6) and Dosing Day 84. Geometric mean ratio for geometric mean titers (Day 84)/(pre-dose) of each group ± 95% confidence interval (CI) are included.

An increase in serotype-specific IgG antibody concentrations, depicted as geometric mean ratios when comparing the pre-dose (Dosing Day 6) to Dosing Day 84, against each of the 13 serotypes, was detected in the animals administered the 13vPnC or 13vPnC MDV (Figure 3) but not in animals administered vehicle control or 13vPnC MDV vehicle controls.

## 4. Discussion

### 4.1. Nonclinical Safety Data in Vaccines Containing 2-PE

The nonclinical safety data for 13vPnC MDV demonstrated that IM administration of up to five doses of 5 mg/dose 2-PE in vaccine formulations is safe and tolerated in a repeat-dose toxicity study. The observed hematological and microscopic findings (minor changes in fibrinogen, injection-site inflammation, and increased splenic germinal centers) were nonadverse and were consistent with an inflammatory and/or immune response to an aluminum-containing vaccine [55]. In addition, the nonclinical safety profile was very similar to that for the 13vPnC single-dose syringe, which does not contain 2-PE.

### 4.2. Other Nonclinical Safety Data on 2-PE

The primary toxicity of 2-PE following oral or dermal exposure is hemolysis, and the available data suggest that the rabbit is the most sensitive nonclinical species [9]. As previously noted, dermal administration of 2-PE results in a much higher bioavailability than that for oral administration in nonclinical species (~78–81% vs. ~0.07–1.6%) [9,10]. Given that the rabbit is the most sensitive species to the hemolytic activity of 2-PE and dermal exposure is a more relevant route to vaccine administration, a previously reported 13-week dermal GLP repeat-dose toxicity study in NZW rabbits serves as a key study for the safety assessment of 2-PE [9,56]. In this study, no treatment-related macroscopic or microscopic changes were observed apart from sporadic and minimal skin irritation; in particular, there was no hematotoxicity [9,56]. As a result, the NOAEL of 2-PE in this study was determined to be 500 mg/kg/day, the highest dose tested.

The developmental toxicity of 2-PE via dermal exposure has also been previously evaluated. In a repeat-dose dermal developmental toxicity study, maternal toxicity was evidenced by the intravascular hemolysis of red blood cells and death in some rabbits in the 600 and 1000 mg/kg/day dose groups [9]. No sign of maternal toxicity was seen at 300 mg/kg/day (NOAEL for maternal toxicity). There was no effect of treatment on the developing fetus up to the highest dose; however, maternal deaths in the high-dose group precluded a full evaluation of developmental toxicity in this group. As a result, the NOAEL for developmental toxicity was 600 mg/kg/day.

Nonclinical and clinical studies evaluating the carcinogenicity, genotoxicity, phototoxicity, and skin sensitization of 2-PE are also available. 2-PE is not genotoxic, carcinogenic, or phototoxic [9]. The prevalence of skin sensitization by 2-PE is low [9].

### 4.3. Clinical Safety Data in Vaccines and Dermal Products Containing 2-PE

2-PE has been included in more than 30 vaccine formulations at amounts ranging from 2.5 to 5 mg per dose with a record of being safe (Table 1, Table 2 and Table 3). Comparable safety profiles and non-inferiority were demonstrated with vaccines formulated with or without 2-PE. A Prev(e)nar13 MDV formulation containing 2-PE at a 4 mg/0.5 mL dose was found safe and non-inferior to the preservative-free single-dose syringe formulation in a clinical trial. In that study, 245 and 244 infants received three doses of MDV or single-dose syringe formulations of 13vPnC respectively, at 2, 3, and 4 months of age [6]. There were no differences in reactogenicity, local or systemic reactions, or severe adverse events between the Prev(e)nar13 MDV and single-dose syringe groups. Similarly, in a clinical study, the reported adverse events for the preservative-free single-dose syringe and the 2-PE-containing MDV (5 mg/dose 2-PE) presentations of Synflorix, a pneumococcal conjugate vaccine, were within similar ranges [57]. In addition, in four clinical trials of Kinrix, a diphtheria, tetanus, and acellular pertussis (DTaP) vaccine, a total of 4013 children were vaccinated with a single dose of Kinrix. Of these, 381 children received a non-U.S. formulation of Kinrix in two clinical trials containing ≤ 2.5 mg 2-PE/0.5 mL dose as a preservative [26]. The safety data for the non-U.S. 2-PE-containing formulation of Kinrix were non-inferior to those for the 2-PE-free formulation. Moreover, in a clinical study, Adacel (3.3 mg/dose 2-PE) immunization of pregnant women in the third trimester was well tolerated and elicited immune responses similar to those in nonpregnant women [11,58]. No Adacel-related adverse pregnancy outcomes were observed. The U.S. CDC currently recommends a single dose of Tdap vaccine for pregnant women during every pregnancy. Altogether, the data indicate that doses of 2-PE up to 3.3 mg administered IM do not present a safety risk for pregnant women [58].

An adverse event potentially related to a vaccine with 2-PE was reported in 1998 [59]. Following a routine administration of the DTaP vaccine Infanrix, which contained a 2.5 mg/dose 2-PE, an 18-month-old infant with a strong family history of allergic reactions, developed two episodes of generalized eczema within 24 h post-vaccination. Irritancy threshold patch testing was positive for 2% 2-PE. This potential toxicity/allergenicity for 2-PE was later addressed by the manufacturer in the regulatory documents of Infanrix Hexa and Infanrix Penta [46,60]. It was concluded that the results of a comparison of the adverse event profiles of a vaccine containing 2-PE and another vaccine without 2-PE gave no reason for concern. In addition, the positive patch rate for 2-PE was reported to be 2%, compared with a 10.7% rate for thimerosal [61], suggesting that systemic allergic reactions during vaccination with 2-PE-containing formulations are probably rare.

Several clinical studies are also available on nonvaccine formulations, such as creams or solutions containing up to 2.2% 2-PE, used in premature neonates and adults [9,62]. The estimated adult daily amounts of 2-PE applied in these studies ranged from 1 to 3.5 g. No adverse effects related to 2-PE were reported in either the pediatric or the adult populations.

## 5. Conclusions

The nonclinical data of the 13vPnC MDV demonstrate that IM administration of up to five doses of 5 mg/dose 2-PE in vaccine formulations is safe in repeat-dose toxicity studies. Moreover, up to four IM doses of 3.3 mg/dose of 2-PE were tolerated and did not result in systemic or developmental toxicity in rabbits, which were identified as the most sensitive nonclinical species for 2-PE toxicity. Hemolysis, the primary toxicity of 2-PE via dermal and oral exposure, was not observed or reported in these IM repeat-dose toxicity studies. The dose in rabbits is approximately 28x higher, on an mg/kg basis (2.5 kg rabbit versus 70 kg human), than that used clinically. Moreover, assuming 100% and ~80% bioavailability for the IM and dermal route of administration, respectively, the rabbit ≥300 mg/kg/day dermal NOAEL is equivalent to ≥240 mg/kg/day if administered via the IM route, which represents a >1678x margin of safety (MoS) over the human dose of 0.143 mg/kg when administered with a vaccine product at up to 20 mg/mL (10 mg/dose) to humans with an average body weight of 70 kg.

2-PE has been included in more than 30 vaccine formulations at amounts ranging from 0.5 to 5 mg per dose with a record of being safe. Comparable safety profiles and non-inferiority have been demonstrated for vaccines formulated with or without 2-PE. Clinical dermal exposure to 2-PE in premature neonates and in adults also showed no 2-PE-related adverse effects.

According to the Opinion of the EU Safety Committee on Consumer Safety, the SCCS recommends the use of a minimum MoS of 25 for adults and 50 for children instead of 100 for 2-PE for its safety assessment, given its favorable nonclinical and clinical safety, and considers 2-PE safe for use as a preservative in cosmetics with a maximum concentration of 1.0% [9]. Therefore, the >1678x MoS of nonclinical (including maternal and developmental) NOAELs, which were identified as daily doses, suggests a negligible risk of 2-PE when given with the vaccines at ≤20 mg/mL (≤10 mg per 0.5 mL dose, 0.143 mg/kg) as a single dose to humans (including pregnant women). Indeed, clinical studies with 2-PE dermal administrations have shown no adverse effects in premature neonates (for the first 7 days of life as a skin disinfectant at 2%) or in adults (for treating wound/burn infections at 2.2% up to 50 mg/kg/day) [9,62].

Given the breadth of clinical and nonclinical data for 2-PE by multiple routes of exposure and following the principle of 3Rs, our risk-assessment-based recommendation is that no additional nonclinical safety studies are needed when evaluating a 2-PE-containing presentation of a previously well-characterized vaccine product if the amount of 2-PE is ≤20 mg/mL or ≤10 mg/dose.

## Figures and Tables

**Table 4:** Group incidences with severities of the 13vPnC-related microscopic observations.

Finding	Male			Female		
	Dosage Group			Dosage Group		
	Saline Control	Vehicle Control	13vPnC	Saline Control	Vehicle Control	13vPnC
Injection Site (Dosing-Phase Necropsy) ^a^	5	5	5	5	5	5
Degeneration/Necrosis, Myofibers	0	1	3	1	1	2
Minimal	0	0	1	0	1	0
Mild	0	1	1	1	0	2
Moderate	0	0	1	0	0	0
Inflammation, Chronic	0	2	4	0	3	5
Minimal	0	0	0	0	1	3
Mild	0	2	2	0	2	0
Moderate	0	0	2	0	0	2
Injection Site (Recovery-Phase Necropsy) ^a^	5	5	5	5	5	5
Degeneration/Necrosis	0	1	2	0	1	3
Minimal	0	1	2	0	1	2
Mild	0	0	0	0	0	1

a. Number examined.

**Table 5:** Group incidences with severities of 13vPnC MDV-related microscopic observations.

Finding	Male			Female		
	Dosage Group			Dosage Group		
	Vehicle Control	13vPnC MDV Vehicle Control	13vPnC MDV	Vehicle Control	13vPnC MDV Vehicle Control	13vPnC MDV
Spleen (Dosing-Phase Necropsy) ^a^	5	5	5	5	5	5
Germinal Centers, Increased						
Minimal	0	2	3	0	0	5
Mild	0	0	1	0	0	0
Spleen (Recovery-Phase Necropsy) ^a^	5	5	5	5	5	5
Germinal Centers, Increased						
Minimal	0	0	2	0	0	3
Intramuscular Site (Dosing-Phase Necropsy) ^a^	5	5	5	5	5	5
Degeneration/Necrosis, Myofibers						
Minimal	3	0	2	2	4	2
Mild	0	0	1	0	0	1
Moderate	1	1	0	0	0	0
Inflammation, Chronic						
Minimal	2	4	2	4	4	4
Mild	1	1	3	1	0	0
Moderate	2	0	0	0	0	1
Intramuscular Site (Recovery-Phase Necropsy) ^a^	5	5	5	5	5	5
Degeneration/Necrosis						
Minimal	1	3	1	1	0	0
Inflammation, Chronic						
Minimal	2	4	3	4	3	4
Mild	2	0	1	0	1	0

a. Number examined.

## Data Availability

The data presented in this study are available in this article.

## Supplementary Materials

The following supporting information can be downloaded at https://www.mdpi.com/article/10.3390/vaccines13050486/s1: Table S1: Hematology parameters; Table S2: Clinical chemistry parameters; Table S3: Tissue collection and organ weights.
