# Supplementary material for: A Similar Nonclinical Safety Evaluation of Prev(e)nar 13 in a Multi-Dose Formulation Containing the Preservative 2-Phenoxyethanol
Source: Vaccines (Basel). 2025 Apr 30;13(5):486. doi: 10.3390/vaccines13050486 (PMC12115998; doi:10.3390/vaccines13050486)
Supplement: Supplementary file 1 [file vaccines-13-00486-s001.zip › vaccines-3558030-supplementary.pdf]

vaccines

MDPI

Supplementary Tables

Supplementary Table S1. Hematology parameters

Hematology and Coagulation Evaluation	
Red blood cell (erythrocyte) count<br>Hemoglobin<br>Hematocrit<br>Mean corpuscular volume<br>Mean corpuscular hemoglobin<br>Mean corpuscular hemoglobin concentration<br>Fibrinogen<br>Prothrombin time	Activated partial thromboplastin time<br>Platelet count<br>White blood cell (leukocyte) count<br>Differential blood cell count<br>Blood smear<br>Reticulocyte count<br>Mean platelet volume<br>Red blood cell distribution width

Supplementary Table S2: Clinical Chemistry Parameters

Clinical Chemistry Evaluation	
Glucose<br>Urea nitrogen<br>Creatinine<br>Total protein<br>Albumin<br>Globulin<br>Albumin/globulin ratio<br>Cholesterol<br>Total bilirubin<br>Alanine aminotransferase<br>Alkaline phosphatase<br>Gamma glutamyltransferase<br>Aspartate aminotransferase<br>Amylase	Lipase<br>Calcium<br>Inorganic phosphorus<br>Sodium<br>Potassium<br>Chloride<br>Triglycerides<br>Creatine kinase<br>Indirect Bilirubin<br>Direct Bilirubin<br>Thyroxine<br>BUN/Creatinine Ratio<br>Lactate Dehydrogenase

 https://doi.org/10.3390/vaccines13050486

Supplementary Table S3: Tissue collection and organ weights

Tissue List	
Aorta<br>Bone Marrow, Sternum and Femur<br>Bone, Sternum<br>**Brain**<br>Cervix<br>**Epididymis**<br>Esophagus<br>Eye<br>**Gall bladder**<br>**Gland, Adrenal**<br>Gland, Mammary<br>Gland, Parathyroid<br>**Gland, Pituitary**<br>**Gland, Prostate**<br>Gland, Salivary<br>Gland, Seminal Vesicle<br>**Gland, Thyroid**<br>Gut-Associate Lymphoid Tissue<br>**Heart**<br>**Kidney**<br>Large Intestine, Cecum<br>Large Intestine, Colon<br>Larynx<br>**Liver**<br>Lung	Joint (distal, femur)<br>Lymph Node, Mandibular<br>Lymph Node, Inguinal<br>Lymph Node, Mesenteric<br>Macroscopic Findings<br>Muscle, Skeletal<br>Nerve, Optic<br>Nerve, Peripheral<br>**Ovary**<br>Oviduct<br>Pancreas<br>Site, Injection<br>Skin<br>Small Intestine, Duodenum<br>Small Intestine, Ileum<br>Small Intestine, Jejunum<br>Spinal Cord<br>**Spleen**<br>Stomach<br>**Testis**<br>**Thymus**<br>Tongue<br>Trachea<br>Urinary Bladder<br>Uterus<br>Vagina
Weights were recorded for bolded organs	
